# Association of activity behaviours and patterns with cardiovascular risk factors in Swiss middle-aged adults: The CoLaus study

**DOI:** 10.1016/j.pmedr.2018.05.012

**Published:** 2018-05-15

**Authors:** Cédric Gubelmann, Panagiotis Antiochos, Peter Vollenweider, Pedro Marques-Vidal

**Affiliations:** a Department of Medicine, Internal Medicine, Lausanne University Hospital, Switzerland; b Heart and Vessels Department, Division of Cardiology, Lausanne University Hospital, Switzerland

**Keywords:** Cardiovascular risk factors, Physical activity, Pattern, Sedentary behaviour, Accelerometry, Epidemiology

## Abstract

The impact of the combination between physical activity (PA) and sedentary (SE) levels on cardiovascular health is poorly known. We assessed the association of activity behaviours and patterns with cardiovascular risk factors in the general population (The CoLaus study, Switzerland, 2014–2017). 2605 adults (54.4% women, age range 45–86 years) had PA and SE levels measured for 14 days using wrist-worn accelerometry. Four activity behaviours: “Couch potato”: low PA & high SE; “Light mover”: low PA & low SE; “Sedentary exerciser”: high PA & high SE, and “Busy bee”: high PA & low SE; and three activity patterns: “Inactive”, “Weekend warrior”, and “Regularly active” were defined. Smoking, obesity, hypertension, dyslipidemia and diabetes were assessed. Relative to ‘Couch potatoes', ‘Sedentary exercisers' and ‘Busy bees' had a lower likelihood of smoking: Odds Ratio (95% confidence interval): 0.40 (0.27–0.61) and 0.62 (0.47–0.81), obesity: 0.43 (0.29–0.63) and 0.41 (0.31–0.54), and diabetes: 0.53 (0.30–0.95) and 0.62 (0.42–0.89), respectively. Relative to ‘Inactives', ‘Weekend warriors' and ‘Regularly actives' had a lower likelihood of smoking: 0.58 (0.43–0.78) and 0.56 (0.44–0.72), obesity: 0.41 (0.30–0.56) and 0.41 (0.32–0.53), hypertension: 0.66 (0.51–0.85) and 0.72 (0.59–0.89), and diabetes: 0.61 (0.38–0.98) and 0.60 (0.42–0.86), respectively. High PA is associated with a favourable cardiovascular risk profile, even when concomitant with high SE or when PA is concentrated on weekends. These findings suggest that being “Sedentary exerciser” or “Weekend warrior” might be sufficient to prevent cardiovascular disease.

## Highlights

• Association of activity behaviours and patterns with cardiovascular risk profile
• 2605 adults from general population assessed by 14-day accelerometry.
• Active individuals had better profile even when highly sedentary.
• Active individuals had better profile even when activity concentrated on weekends.
• Sedentary exerciser, Weekend warrior may be enough for cardiovascular prevention.

## 1 Introduction

The beneficial effect of regular physical activity (PA) on cardiovascular disease (CVD) is well established (Li and Siegrist, 2012; Wasfy and Baggish, 2016). Beyond the dose-response effect, other components of PA have been shown to impact cardiovascular (CV) health: (i) its combination with sedentary (SE) levels (i.e. “activity behaviour”) as described by Bakrania and al. (Bakrania et al., 2016); and (ii) its distribution over time (i.e. “activity pattern”) as described by Lee et al. (2004) and O'Donovan et al. (2017)). Indeed, the benefits of PA could be altered either by a high SE level (Buman et al., 2014; Sugiyama et al., 2008), or by exercising only 1–2 times per week (Lee et al., 2004).

Part of the effect of PA on CVD is mediated through changes in cardiovascular risk factors (CVRF) (Mora et al., 2007). High PA levels are associated with lower levels of body mass index (BMI), blood pressure (BP), lipids and glycaemia (Wasfy and Baggish, 2016; Shuval et al., 2014). Paradoxically, several studies reported no association between SE levels and CVRF (Shuval et al., 2014; Saunders et al., 2013) but those findings have been questioned (Healy et al., 2008; Qi et al., 2015; Healy et al., 2011). These contradictory findings are likely due to the fact that most studies focused separately on SE or on PA but not on their combinations. Indeed, a recent meta-analysis (Ekelund et al., 2016) described an interaction between PA and SE, showing that high PA levels could attenuate the deleterious effect of SE. Hence, analysis of PA and SE combinations seems necessary to provide more valuable information with regards to their association with CVRF, and thus with CVD risk.

Nevertheless, to date, little is known on the association of activity behaviours and patterns with CVRF. The existent literature is limited as: (i) it did not take into account all traditional CVRF (Bakrania et al., 2016; Loprinzi et al., 2014); (ii) the definition of behaviours (Sugiyama et al., 2008; Cristi-Montero et al., 2017) or patterns (Lee et al., 2004; O'Donovan et al., 2017) relied on self-reported data, or (iii) it did not adjust for major confounders such as age, gender or socio-economic factors (Lee et al., 2004; O'Donovan et al., 2017).

Therefore, this study aimed to assess the association of activity behaviours and patterns with traditional CVRF in a population-based sample aged 45–86 years from the city of Lausanne, Switzerland (CoLaus study).

## 2 Methods

### 2.1 Recruitment of participants

The detailed description of the recruitment of the CoLaus study and the follow-up procedures has been described previously (Firmann et al., 2008; Marques-Vidal et al., 2011). Briefly, the CoLaus study is a population-based cohort exploring the biological, genetic and environmental determinants of CVD. A non-stratified, representative sample of the population of Lausanne (Switzerland) was recruited between 2003 and 2006 based on the following inclusion criteria: (i) age 35–75 years and (ii) willingness to participate. The second follow-up occurred ten years after the baseline survey: 2605 subjects participated in an optional module assessing their PA levels for 14 days and were sufficiently studied to be included in the analysis (see exclusion criteria). For this study, we performed a cross-sectional analysis using data of the second follow-up only.

### 2.2 Physical activity measurement

PA was assessed using a wrist-worn triaxial accelerometer (*GENEActiv*, Activinsights Ltd., United Kingdom). This device has been validated against reference methods (Esliger et al., 2011). The intra- and inter-instrument coefficients of variation were 1.4% and 2.1%; and the correlations with methods such as mechanical shaking and indirect calorimetry were strong (r = 0.98 and r = 0.83) (Esliger et al., 2011). The accelerometers were pre-programmed with a 50 Hz sampling frequency, and subsequently attached to the participants' right wrist irrespective of their dominant wrist (Dieu et al., 2017). To optimally capture PA gradient between week and weekend days, participants were requested to wear the device continuously, day and night, for 14 days in their free-living conditions.

Accelerometry data were downloaded using the *GENEActiv* software version 2.9 (*GENEActiv*, Activinsights Ltd., United Kingdom) and transformed into 60-s epoch files. Data were analyzed using the *GENEActiv macro file* ‘General physical activity’ version 1.9 (GENEActiv, 2014) which is based on validated intensity cutoffs (Esliger et al., 2011): SE (<241 g·min), light intensity PA (241–338 g·min) and moderate-to-vigorous PA (>338 g·min). Conversely, no information was available regarding the criteria used for sleep and non-wear time (proprietary). A valid day was defined as ≥10 h (i.e. 600 min) of diurnal wear-time. For each participant, the time (in minutes) spent in light intensity PA, moderate-to-vigorous intensity PA (MVPA) and in SE was averaged for all valid days and separately for valid week and weekend days. At least 5 week days and 2 weekend days of valid accelerometry data were required (see exclusion criteria).

### 2.3 Activity behaviours

**Fig. 1:**
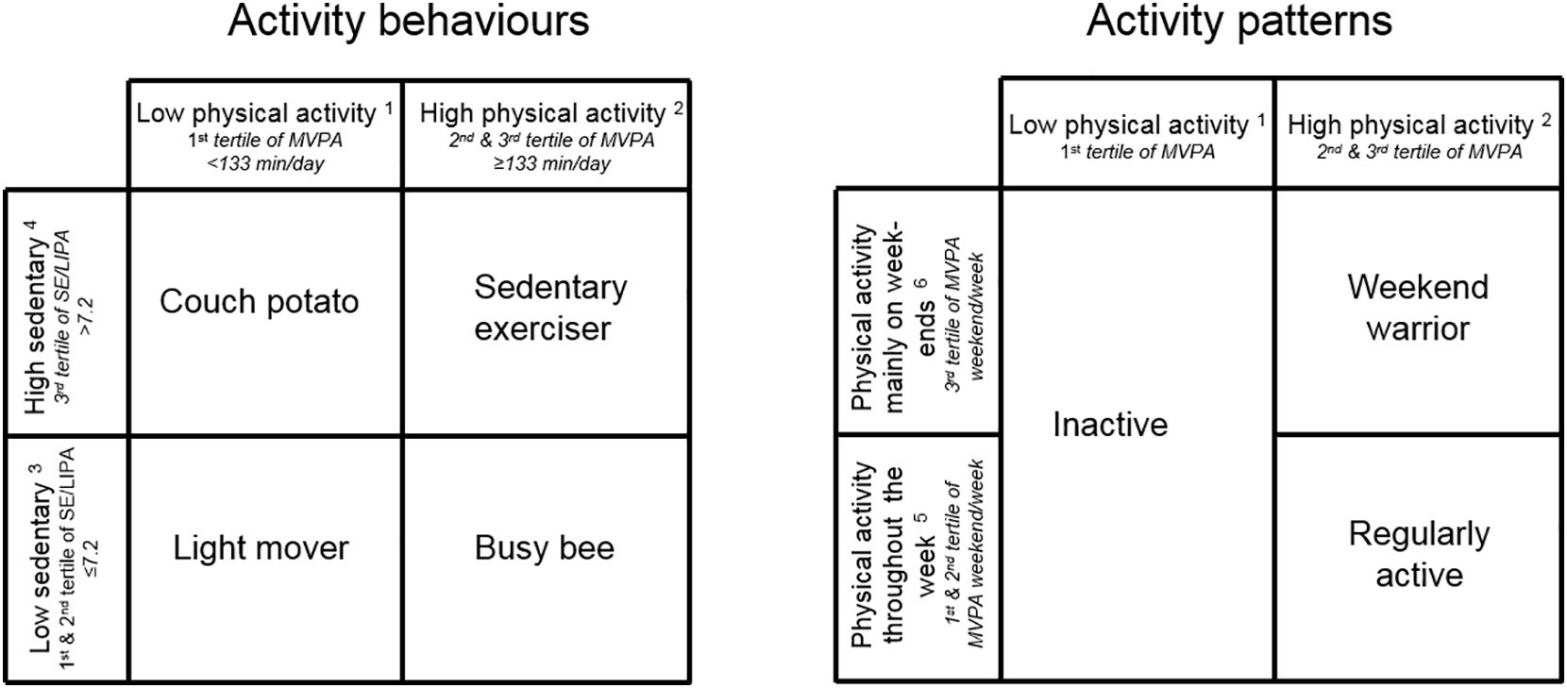
Mutually exclusive activity behaviours and patterns. The CoLaus study, Switzerland, 2014–2017. ^1^ tertile 1 of average moderate-to-vigorous physical activity time; ^2^ tertile 2 or 3 of average moderate-to-vigorous physical activity time; ^3^ tertiles 1 or 2 of the ratio between average sedentary time and average light physical activity time; ^4^ tertiles 3 of the ratio between average sedentary time and light physical activity time; ^5^ tertiles 1 or 2 of the ratio between average moderate-to-vigorous physical activity time on weekend days and average moderate-to-vigorous physical activity time on week days. ^6^ tertile 3 of the ratio between average moderate-to-vigorous physical activity time on weekend days and average moderate-to-vigorous physical activity time on week days.

Activity behaviours were defined according to the combination between PA and SE status. For PA status, participants were split into tertiles of average MVPA time and classified as “low PA” if they were in the first tertile (<133 min/day) and as “high PA” otherwise. Previous studies have shown that light intensity PA could influence CV health (Buman et al., 2014). SE status was defined according to the ratio between the average SE time and the average light intensity PA time as performed by others (Bakrania et al., 2016; Loprinzi et al., 2014). Participants were classified as “high SE” if they were in the highest tertile (>7.2) and as “low SE” otherwise. This allowed creating four mutually exclusive activity behaviours (Fig. 1): 1) “Couch potato”: “low PA” & “high SE”; 2) “Light mover”: “low PA” & “low SE”; 3) “Sedentary Exerciser”: “high PA” & “high SE”; and 4) “Busy bee”: “high PA” & “low SE”.

### 2.4 Activity patterns

Activity patterns were defined according to PA status and its distribution throughout the week. For PA status, participants were classified as “low PA” if they were in the first tertile of average MVPA time (<133 min/day). For the distribution of PA, average MVPA time on weekend days was divided by average MVPA time on week days and split into tertiles. Participants were categorized as ‘PA mainly on weekends’ if they were in the highest tertile and as ‘PA throughout the week’ otherwise. This classification allowed creating three mutually exclusive activity patterns (Fig. 1): 1) “Inactive”: “low PA”; 2) “Weekend warrior”: “high PA” & ‘PA mainly on weekends’; and 3) “Regularly active”: “high PA” & ‘PA throughout the week’.

### 2.5 Cardiovascular risk factors

CVRF were assessed at second follow-up, when PA was measured.

Smoking status was collected by questionnaire. Participants were considered as smokers if they reported current smoking (any type of tobacco combustion) and non-smokers otherwise.

Body weight and height were measured to the nearest 0.1 kg and 5 mm (Seca® scale, Seca® height gauge, Hamburg, Germany), with participants in light indoor clothes standing without shoes. Body mass index (BMI) was computed as weight/height^2^. Obesity was defined by a BMI ≥30 kg/m^2^.

In accordance with US recommendations (Pickering et al., 2005), blood pressure (BP) was measured three times using an Omron® HEM-907 automated oscillometric sphygmomanometer after at least 10 min' rest in a seated position and the average of the last two measurements was used. Hypertension was defined as a systolic BP ≥140 mmHg and/or a diastolic BP ≥90 mmHg and/or if the participant reported having an anti-hypertensive treatment.

A fasting venous blood sample was drawn and measurements performed by the clinical laboratory of the Lausanne university hospital. CVRF included glucose, and LDL-cholesterol that was calculated using the Friedewald formula if triglycerides were <4.6 mmol/l. Diabetes was defined by a fasting glucose ≥7.0 mmol/l and/or if the participant reported having an anti-diabetic treatment. Dyslipidemia was defined either by using the LDL-cholesterol thresholds according to the PROspective CArdiovascular Münster (PROCAM) risk score (Assmann et al., 2007) adapted for Switzerland (Moser et al., 2014), or if the participant reported having a lipid lowering treatment. Although HDL-cholesterol and triglycerides can also be influenced by PA, they were not considered as only LDL-cholesterol is used in the Swiss definition of dyslipidemia (Moser et al., 2014).

### 2.6 Socio-economic data

Demographic, professional occupation and household income data were collected by questionnaire. Monthly household income before social charges was expressed in Swiss francs (1 CHF = 1.012 US$ or 0.913 € as of 16 May 2017). Educational level was collected at baseline by questionnaire and categorized as low (obligatory school or apprenticeship), medium (high school), or high (university degree).

### 2.7 Exclusion criteria

**Fig. 2:**
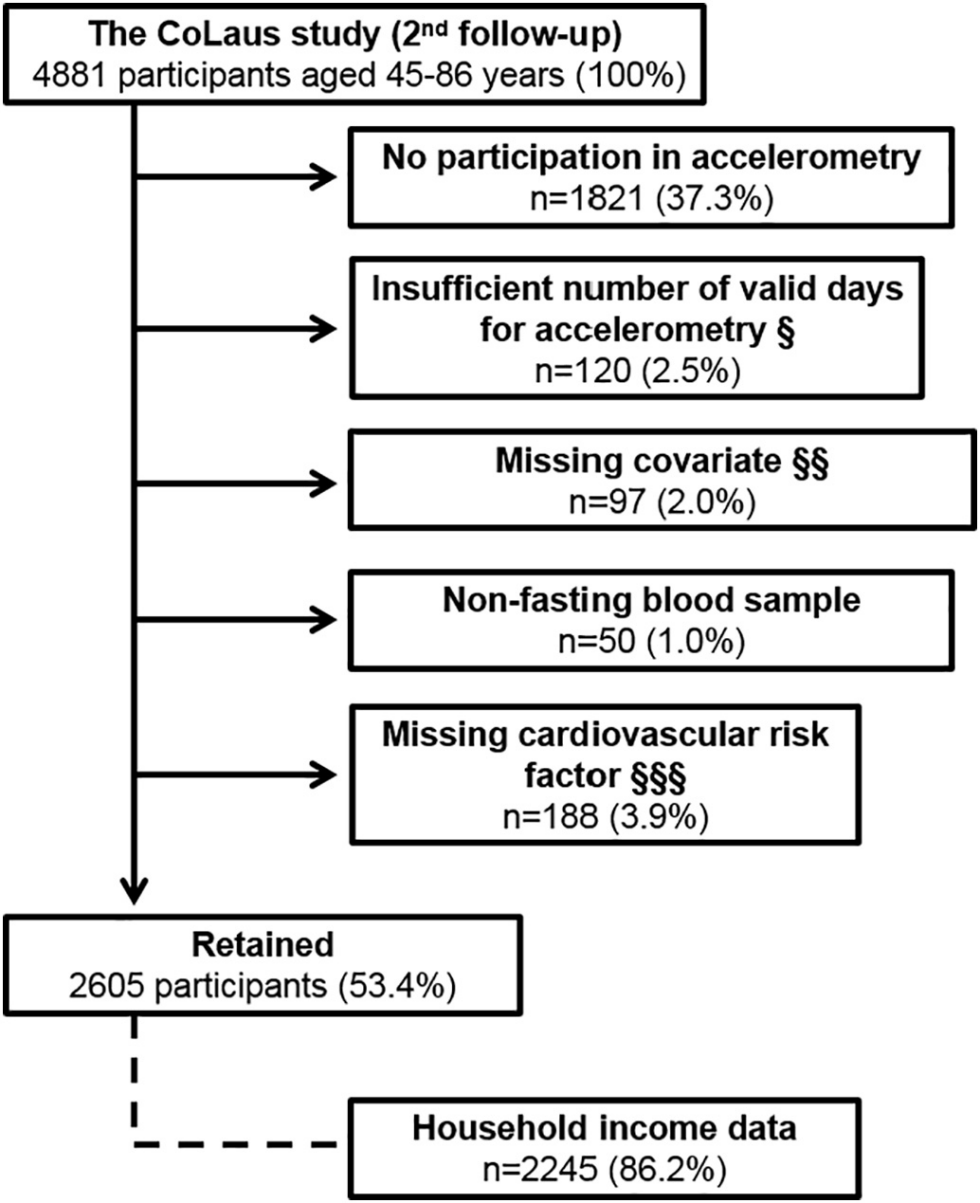
Selection procedure. The CoLaus study, Switzerland, 2014–2017. §: <5 week days or <2 weekend days with minimum 10 h of diurnal wear-time. §§: any missing data in professional occupation, educational level, or body mass index. §§§: any missing data in smoking, obesity, hypertension, dyslipidemia or diabetes. Percentages were calculated using the total sample size as denominator.

Participants were excluded if they: (i) did not participate in accelerometry; (ii) had <5 weekdays or 2 weekend days of valid accelerometry data, (iii) had missing data for covariates (professional occupation, educational level, or body mass index), (iv) were non-fasting, or (v) had missing data in CVRF (Fig. 2).

### 2.8 Statistical analysis

Statistical analyses were conducted using Stata version 14.0 for windows (Stata Corp, College Station, Texas, USA). Results were expressed as number of participants (percentage) for categorical variables or as average ± standard deviation for continuous variables. Between-group comparisons were performed using chi-square and one-way analysis of variance for categorical and continuous variables, respectively.

Multivariate analyses were conducted using logistic regression with CVRF as the dependent variable. All multivariate models were adjusted for age (continuous), gender (male/female), professional occupation (no/yes), educational level (high/medium/low), and accelerometer diurnal wear-time (continuous); with an additional adjustment on BMI for the associations with hypertension, dyslipidemia and diabetes. Analyses were further adjusted for household income. Finally, several sensitivity analyses were performed: (i) using medians instead of tertiles for the definition of activity behaviours and patterns; (ii) by excluding participants with history of CVD; (iii) by including all participants irrespective of missing data in CVRF; or (iv) without adjustment for BMI. Results were expressed as odds ratio and 95% confidence interval. Statistical significance was assessed for a two-sided test with p < 0.05. As this was mainly an exploratory analysis, we decided not to adjust for multiple comparisons in order to capture any potential interesting association.

### 2.9 Ethical statement and consent

The Ethics Commission of Canton Vaud approved the second follow-up of the CoLaus study (reference (Aune et al., 2015)/14, decision of 11th March 2014). The study was performed in agreement with the Helsinki declaration and in accordance with the applicable Swiss legislation. All participants gave their signed informed consent before entering the study.

## 3 Results

### 3.1 Selection procedure and characteristics of excluded and included participants

The selection procedure is indicated in Fig. 2. Of the initial 4881 participants, 2605 (53.4%) were retained for analysis. Included and excluded participants' characteristics are presented in Supplementary Table 1. Included participants were younger, less likely smoking, more prone to have a professional occupation, a higher educational level or household income, and had lower accelerometer diurnal wear-time than excluded ones; they had also a lower CV risk (PROCAM), and lower prevalences of obesity, hypertension, diabetes and dyslipidemia. Among included participants, average time (±standard deviation) of accelerometer diurnal wear on valid days was 15.4 ± 1.1 h. The number of valid accelerometry days was 9.3 ± 1.2 on weekdays and 3.7 ± 0.7 on weekends (mean ± standard deviation).

### 3.2 Association of activity behaviours with cardiovascular risk factors

Of the final 2605 participants, 545 (20.9%) were categorized as “Couch potatoes”, 306 (11.8%) as “Light movers”, 321 (12.3%) as “Sedentary Exercisers”, and finally 1433 (55.0%) as “Busy bees”. The “Light movers” and “Busy bees” were more frequently female (Supplementary Table 2).

The bivariate associations between activity behaviours and CVRF are described in Supplementary Tables 2 while the multivariate analyses are presented in Table 1, Table 2. On bivariate analysis, the “Sedentary exerciser” and “Busy bee” behaviours were related to lower rates to smoke, obesity, hypertension, dyslipidemia and diabetes, compared to the “Couch potatoes”. The “Light movers” presented higher rates of dyslipidemia. After multivariate adjustment, all associations remained excepted that the “Sedentary exerciser” and “Busy bee” behaviours were no longer associated with dyslipidemia, and only non-significant trends persisted between the “Light movers” and higher rates of hypertension (p = 0.10) and between the “Sedentary exercisers” and lower rates of hypertension (p = 0.11) (Table 1). Additional adjustment for household income lead mostly to similar findings (Table 2). The “Busy bees” were negatively associated with smoking, obesity, hypertension and diabetes. It was similar for the “Sedentary exercisers” but only a non-significant trend was found with lower rates of diabetes (p = 0.08). Furthermore, a non-significant trend persisted between the “Light movers” and higher rates of dyslipidemia (p = 0.28). Most associations remained in sensitivity analyses (Supplementary Tables 4–7).

**Table 1:** Multivariate analysis of the cardiovascular risk factors associated with activity behaviours and patterns. The CoLaus study, Switzerland, 2014–2017.

	Smoking	Obesity	Hypertension ^1^	Dyslipidemia ^1^	Diabetes ^1^
Activity behaviours					
Couch potato	1 (ref)	1 (ref)	1 (ref)	1 (ref)	1 (ref)
Light mover	1.03 (0.72–1.46)	1.00 (0.72–1.39)	1.31 (0.95–1.80)	**1.44 (1.05–1.97)**	0.97 (0.63–1.50)
Sedentary exerciser	**0.40 (0.27–0.61)**	**0.43 (0.29–0.63)**	0.77 (0.56–1.06)	1.09 (0.79–1.52)	**0.53 (0.30–0.95)**
Busy bee	**0.62 (0.47–0.81)**	**0.41 (0.31–0.54)**	**0.77 (0.61–0.98)**	1.07 (0.84–1.36)	**0.62 (0.42–0.89)**
Activity patterns					
Inactive	1 (ref)	1 (ref)	1 (ref)	1 (ref)	1 (ref)
Weekend warrior	**0.58 (0.43–0.78)**	**0.41 (0.30–0.56)**	**0.66 (0.51–0.85)**	0.90 (0.69–1.18)	**0.61 (0.38–0.98)**
Regularly active	**0.56 (0.44–0.72)**	**0.41 (0.32–0.53)**	**0.72 (0.59–0.89)**	0.95 (0.77–1.18)	**0.60 (0.42–0.86)**

Results are expressed as odds ratio (OR) and (95% confidence interval). Statistical analyses performed by logistic regressions adjusted for age, gender, professional occupation, educational level and accelerometer diurnal wear-time; with a further adjustment on body mass index ^1^. Significant (p < 0.05) odds ratio are indicated in bold.

**Table 2:** Multivariate analysis of the cardiovascular risk factors associated with activity behaviours and patterns, with adjustment on household income. The CoLaus study, Switzerland, 2014–2017.

	Smoking	Obesity	Hypertension ^1^	Dyslipidemia ^1^	Diabetes ^1^
Activity behaviours					
Couch potato	1 (ref)	1 (ref)	1 (ref)	1 (ref)	1 (ref)
Light mover	1.03 (0.70–1.51)	0.92 (0.64–1.32)	1.22 (0.86–1.72)	1.24 (0.87–1.75)	1.06 (0.66–1.71)
Sedentary exerciser	**0.37 (0.24–0.58)**	**0.48 (0.31–0.73)**	**0.69 (0.49–0.97)**	0.95 (0.67–1.35)	0.58 (0.32–1.06)
Busy bee	**0.62 (0.46–0.82)**	**0.45 (0.33–0.60)**	**0.73 (0.57–0.94)**	1.05 (0.81–1.37)	**0.63 (0.42–0.95)**
Activity patterns					
Inactive	1 (ref)	1 (ref)	1 (ref)	1 (ref)	1 (ref)
Weekend warrior	**0.54 (0.40–0.75)**	**0.49 (0.36–0.69)**	**0.62 (0.47–0.81)**	0.85 (0.64–1.13)	0.64 (0.39–1.06)
Regularly active	**0.57 (0.43–0.74)**	**0.45 (0.35–0.59)**	**0.70 (0.56–0.88)**	1.00 (0.80–1.26)	**0.59 (0.40–0.87)**

Results are expressed as odds ratio (OR) and (95% confidence interval). Statistical analyses performed by logistic regressions adjusted for age, gender, professional occupation, educational level, household income, and accelerometer diurnal wear-time; with a further adjustment on body mass index ^1^. Significant (p < 0.05) odds ratio are indicated in bold.

### 3.3 Association of activity patterns with cardiovascular risk factors

Of the final 2605 participants, 851 (32.7%) were categorized as “Inactives”, 592 (22.7%) as “Weekend warriors”, and finally 1162 (44.6%) as “Regularly actives”. The “Weekend warriors” and “Regularly actives” were more frequently female (Supplementary Table 3).

The bivariate associations between activity patterns and CVRF are described in Supplementary Table 3 and the multivariate analyses are presented in Table 1, Table 2. On bivariate analysis, the “Weekend warrior” and “Regularly active” patterns were related to lower rates of smoking, obesity, hypertension, dyslipidemia and diabetes, compared to the “Inactives”. After multivariate adjustment, all associations remained excepted that the “Weekend warrior” and “Busy bee” patterns were no longer related to dyslipidemia (Table 1). Results did not change after additional adjustment for household income excepted that only a non-significant trend persisted between the “Weekend warrior” and lower rates of diabetes (p = 0.09) (Table 2). Most associations remained in sensitivity analyses (Supplementary Tables 4–7). It is to note that without adjustment for BMI the “Weekend warrior” and “Regularly active” patterns were negatively associated with dyslipidemia (Supplementary Table 7).

## 4 Discussion

This study assessed the association of PA and SE behaviours and patterns with traditional CVRF using a 14-day accelerometry measurement in a population-based setting. Our results indicate that, among activity behaviours, the “Busy bees” and “Sedentary exercisers” are associated to a lower prevalence of CVRF whereas no association was found for the “Light movers”. Similarly, among activity patterns, the “Regularly actives” and “Weekend warriors” were related to lower prevalence of CVRF. Thus, adopting sufficient PA despite high SE levels or concentrating PA on weekends might be enough to prevent CVD.

### 4.1 Activity behaviours

The “Sedentary exerciser” and “Busy bee” behaviours were negatively associated with smoking whereas no association was found for the “Light movers”. These findings are partly in agreement with Bakrania et al. (2016) that demonstrated lower prevalence rates of smoking among the “Sedentary exercisers” but higher ones for the “Busy bees” and the “Light movers”; but these results were not adjusted for potential confounders. Overall, PA has been negatively associated with smoking Hassandra et al. (2015). The “Sedentary exercisers” and “Busy bees” were also negatively associated with obesity whereas no association was found for the “Light movers”, a finding in agreement with other studies (Bakrania et al., 2016; Loprinzi et al., 2014; Cristi-Montero et al., 2017) but not with another one (Sugiyama et al., 2008) showing also lower prevalence rates of obesity among the “Light movers”. This discrepancy is possibly due to the fact that they restricted their analysis to leisure-time PA, therefore misclassifying active workers as “Light movers”. Finally, both “Sedentary exerciser” and “Busy bee” behaviours were negatively associated with hypertension whereas a non-significant positive trend was found for the “Light movers”, a finding in agreement with another study (Cristi-Montero et al., 2017). Finally, our results suggest that individuals adopting high PA levels are less prone to smoke and less likely obese or hypertensive, independently of their SE levels.

The “Sedentary exerciser” and “Busy bee” behaviours showed no association with dyslipidemia. “Light movers” had higher prevalence rates of dyslipidemia relative to “Couch potatoes”, but this association was no longer significant after full adjustment. These findings are in agreement with previous studies (Loprinzi et al., 2014; Cristi-Montero et al., 2017), and with the fact that PA (Wasfy and Baggish, 2016) and SE (Qi et al., 2015) do not significantly alter LDL-cholesterol levels.

The “Busy bees” and “Sedentary exercisers” were negatively associated with diabetes whereas no association was found for the “Light movers”. Whether activity behaviours are associated with diabetes is still debated. A recent study showed lower likelihoods of diabetes among the “Busy bees”, “Sedentary exercisers” and “Light movers” (Cristi-Montero et al., 2017) while Bakrania et al. (2016) showed lower glycated haemoglobin levels only among the “Busy bees” and “Sedentary exercisers”. Another study reported no association with glycaemia (Loprinzi et al., 2014). Discrepancies with our results are possibly due to the fact that: 1) they used self-reported PA and SE (Cristi-Montero et al., 2017); or 2) they took continuous markers of diabetes with no threshold allowing the distinction between diabetic and non-diabetic participants (Bakrania et al., 2016; Loprinzi et al., 2014) . Finally, our results suggest that adopting low SE levels might be necessary for PA to be beneficial on glucometabolism but it should be further explored.

### 4.2 Activity patterns

The “Weekend warrior” and “Regularly active” patterns were related to lower prevalence rates of smoking, a finding in agreement with other studies (Lee et al., 2004; O'Donovan et al., 2017). They were also related to lower prevalence rates of obesity but it remains a matter of debate in literature: a study reported slightly higher BMI levels among the “Weekend warriors” (Lee et al., 2004) while another reported no difference (O'Donovan et al., 2017); however, none of these contradictive findings adjusted for potential confounders. The “Weekend warriors” and “Regularly actives” were related to lower prevalence rates of hypertension, which is in agreement with a previous study (Lee et al., 2004). Finally, our results suggest that individuals with high PA levels are less likely to smoke, and less prone to be obese or hypertensive, independently of PA distribution.

In our study, no association remained between activity patterns and dyslipidemia after adjustment for BMI. This observation was contradicted by a previous study showing a slightly lower prevalence of self-reported dyslipidemia among the “Weekend warriors” (Lee et al., 2004); however this contradictory study did not adjust for potential confounders. Finally, our results suggest that the effect of PA on dyslipidemia is mediated by changes in BMI.

The “Weekend warriors” and “Regularly actives” were related to lower prevalence rates of diabetes whereas no association was found for the “Light movers”. High PA levels protect against diabetes, mainly due to an increase in glucose transporters (GLUT4) (Aune et al., 2015). Interventional studies also indicated that regular PA (≥3 days per week) is associated with improved insulin sensitivity and glycaemic control (Bird and Hawley, 2016). Our results confirm these findings at a population level, and further suggest that concentrating PA on weekends also exert a beneficial effect on glucometabolism. These findings should be confirmed in longitudinal studies exploring the effect of activity patterns on incident impaired fasting glucose or diabetes.

### 4.3 Study strengths and limitations

As far as we know, this is the first study exploring the association of both activity behaviours and patterns with CVRF. Importantly, and contrary to recent findings (Bakrania et al., 2016; O'Donovan et al., 2017; Cristi-Montero et al., 2017), PA and SE were objectively assessed and the analyses included all traditional CVRF.

This study also has several limitations. Firstly, the cross-sectional design of our study precludes the assessment of any causal effect of activity behaviours and patterns on CVRF; the next follow-up of the CoLaus participants will enable assessing causal effects. Secondly, the accelerometer was worn on the right wrist. Although it might be more prone to noisy movements, previous findings found no impact on PA assessment (Esliger et al., 2011; Dieu et al., 2017). Thirdly, *GENEActiv* accelerometers have been suggested to over-report MVPA (Rosenberger et al., 2016); still, as MVPA levels were categorized into tertiles and not absolute values this should not impact the validity of our results. Fourthly, it was not possible to know how accelerometer non-wear time was computed, as the algorithm was proprietary and the *GENEActiv* company did not provide it. Fifthly, the definition of dyslipidemia has been developed for the Swiss population; therefore, our findings might not be generalizable to other countries. Sixthly, as the Swiss definition for dyslipidemia (Moser et al., 2014) is limited to ages <75 years, participants older than 75 had their risk calculated using 75 years instead of their real age. This could underestimate the prevalence of dyslipidemia in this age group. Finally, included participants had lower CV risks and higher socio-economic levels than excluded ones. This is a common selection bias also observed in other large epidemiological studies using accelerometry (Hassani et al., 2014; Loprinzi et al., 2013), and it would be interesting that our findings be replicated in other cohorts with a different socioeconomic background.

## 5 Conclusion

In a population-based sample aged 45 to 86 years, high PA levels are associated with a favourable CV risk profile, even in presence of high SE levels or when PA is concentrated on weekends. Thus, being a “Sedentary exerciser” or a “Weekend warrior” might be enough to prevent CVD.

## Appendix A Supplementary data

Supplementary tables Image 1
