# Supplementary material for: Association of activity behaviours and patterns with cardiovascular risk factors in Swiss middle-aged adults: The CoLaus study
Source: Prev Med Rep. 2018 May 15;11:31–6. doi: 10.1016/j.pmedr.2018.05.012 (PMC6030388; doi:10.1016/j.pmedr.2018.05.012)
Supplement: Supplementary file 1 — Supplementary tables [file mmc1.docx]

**Supplementary table 1:** Characteristics of excluded and included participants. The CoLaus study, Switzerland, 2014-2017.

|  | **Included** | **Excluded** | **P-value** |
| --- | --- | --- | --- |
| Sample size | 2605 | 2276 |  |
| Age (years) | 61.8 ± 9.9 | 64.2 ± 10.9 | <0.01 |
| Female | 54.4 | 55.9 | 0.27 |
| Professional occupation | 57.2 | 47.4 | <0.01 |
| Educational level |  |  | 0.02 |
| High | 22.3 | 19.8 |  |
| Medium | 26.4 | 25.1 |  |
| Low | 51.3 | 55.1 |  |
| Household income ^1^ |  |  | <0.01 |
| <5000 CHF | 25.2 | 31.1 |  |
| 5000-9499 CHF | 43.4 | 43.8 |  |
| >9499 CHF | 31.4 | 25.1 |  |
| Smoking | 17.2 | 21.5 | <0.01 |
| Cardiovascular risk (PROCAM) |  |  | <0.01 |
| Very low | 63.8 | 57.9 |  |
| Low | 20.5 | 24.5 |  |
| Intermediate | 11.1 | 12.6 |  |
| High | 4.6 | 5.1 |  |
| High physical activity | 67.3 | 62.9 | 0.06 |
| Average MVPA time (min/day) | 178.3 ± 85.8 | 171.1 ± 95.3 | 0.11 |
| Low sedentary | 72.1 | 67.8 | 0.09 |
| Average sedentary time (min/day) | 636.6 ± 105.2 | 622.1 ± 116.6 | 0.01 |
| Average LIPA time (min/day) | 109.1 ± 33.7 | 107.5 ± 36.6 | 0.36 |
| Accelerometer diurnal wear-time (hour/day) | 15.4 ± 1.1 | 15.0 ± 1.4 | <0.01 |
| Obesity | 17.5 | 20.9 | <0.01 |
| Body mass index (kg/m^2^) | 26.3 ± 4.6 | 26.6 ± 4.8 | 0.02 |
| Hypertension | 43.1 | 54.8 | <0.01 |
| Systolic blood pressure (mmHg) | 125.7 ± 17.4 | 128.5 ± 18.5 | <0.01 |
| Diastolic blood pressure (mmHg) | 77.2 ± 10.5 | 77.7 ± 10.8 | 0.11 |
| Diabetes | 9.0 | 13.5 | <0.01 |
| Fasting glucose (mmol/l) | 5.4 ± 1.0 | 5.5 ± 1.2 | <0.01 |
| Dyslipidemia | 36.2 | 43.6 | <0.01 |
| LDL-cholesterol (mmol/l) | 3.2 ± 0.9 | 3.1 ± 0.9 | 0.10 |

LIPA, light intensity physical activity; MVPA, moderate-to-vigorous intensity physical activity. ^1^ 1 CHF=1.012 US$ or 0.913 € as of 16 May 2017. Results are expressed as mean ± standard deviation or as percentage. Statistical analyses by chi-square and Student t-test.

**Supplementary table 2:** Characteristics of participants, by activity behaviours. The CoLaus study, Switzerland, 2014-2017.

|  | ***Couch potato*** | ***Light mover*** | ***Sedentary exerciser*** | ***Busy bee*** | **P-value** |
| --- | --- | --- | --- | --- | --- |
| Sample size | 545 (20.9%) | 306 (11.8%) | 321 (12.3%) | 1433 (55.0%) |  |
| Age (years) | 65.6 ± 10.6 | 66.4 ± 10.1 | 58.2 ± 8.7 | 60.2 ± 9.0 | <0.01 |
| Female | 37.4 | 62.1 | 42.4 | 61.8 | <0.01 |
| Professional occupation | 41.1 | 43.5 | 71.0 | 63.2 | <0.01 |
| Educational level |  |  |  |  | <0.01 |
| High | 26.8 | 17.7 | 30.8 | 19.7 |  |
| Medium | 26.8 | 24.8 | 24.9 | 26.9 |  |
| Low | 46.4 | 57.5 | 44.2 | 53.4 |  |
| Household income ^1^ |  |  |  |  | <0.01 |
| <5000 CHF | 25.8 | 39.5 | 16.4 | 24.1 |  |
| 5000-9499 CHF | 43.1 | 39.8 | 36.9 | 45.8 |  |
| >9499 CHF | 31.1 | 20.7 | 46.7 | 30.1 |  |
| Smoking | 20.6 | 21.2 | 11.8 | 16.3 | <0.01 |
| Obesity | 25.9 | 26.8 | 13.1 | 13.4 | <0.01 |
| Hypertension | 57.3 | 61.1 | 35.8 | 35.5 | <0.01 |
| Dyslipidemia | 46.1 | 51.3 | 30.8 | 30.4 | <0.01 |
| Diabetes | 17.4 | 14.7 | 5.6 | 5.4 | <0.01 |

^1^ 1 CHF=1.012 US$ or 0.913 € as of 16 May 2017. Results are expressed as mean ± standard deviation or as percentage. Statistical analyses by chi-square and one-way analysis of variance, comparing activity behaviours.

**Supplementary table 3:** Characteristics of participants, by activity patterns. The CoLaus study, Switzerland, 2014-2017

|  | ***Inactive*** | ***Weekend warrior Sed*** | ***Regularly active*** | **P-value** |
| --- | --- | --- | --- | --- |
| Sample size | 851 (32.7%) | 592 (22.7%) | 1162 (44.6%) |  |
| Age (years) | 65.8 ± 10.4 | 58.5 ± 8.7 | 60.5 ± 9.1 | <0.01 |
| Female | 53.7 | 40.5 | 42.3 | <0.01 |
| Professional occupation | 42.0 | 74.2 | 59.8 | <0.01 |
| Educational level |  |  |  | <0.01 |
| High | 23.5 | 30.9 | 17.0 |  |
| Medium | 26.1 | 29.4 | 25.1 |  |
| Low | 50.4 | 39.7 | 57.8 |  |
| Household income ^1^ |  |  |  | <0.01 |
| <5000 CHF | 30.6 | 18.0 | 25.1 |  |
| 5000-9499 CHF | 42.0 | 39.1 | 46.8 |  |
| >9499 CHF | 27.4 | 42.9 | 28.2 |  |
| Smoking status | 20.8 | 16.1 | 15.2 | <0.01 |
| Obesity | 26.2 | 12.2 | 13.9 | <0.01 |
| Hypertension | 58.6 | 31.1 | 37.9 | <0.01 |
| Dyslipidemia | 47.9 | 26.2 | 32.7 | <0.01 |
| Diabetes | 16.5 | 4.6 | 5.9 | <0.01 |

^1^ 1 CHF=1.012 US$ or 0.913 € as of 16 May 2017. Results are expressed as mean ± standard deviation or as percentage. Statistical analyses by chi-square and one-way analysis of variance, comparing activity patterns.

**Supplementary table 4:** Multivariate analysis of the cardiovascular risk factors associated with activity behaviours and patterns defined using medians. The CoLaus study, Switzerland, 2014-2017.

|  | **Smoking** | **Obesity** | **Hypertension ^1^** | **Dyslipidemia ^1^** | **Diabetes ^1^** |
| --- | --- | --- | --- | --- | --- |
| **Activity behaviours** |  |  |  |  |  |
| *Couch potato* | 1 (ref) | 1 (ref) | 1 (ref) | 1 (ref) | 1 (ref) |
| *Light mover* | 1.14 (0.80 - 1.63) | 0.89 (0.63 - 1.26) | **1.38 (1.01 - 1.90)** | 1.06 (0.77 - 1.45) | 1.26 (0.79 - 2.01) |
| *Sedentary exerciser* | **0.52 (0.35 - 0.77)** | **0.51 (0.35 - 0.76)** | **0.69 (0.51 - 0.94)** | 0.90 (0.66 - 1.24) | 0.65 (0.36 - 1.20) |
| *Busy bee* | **0.73 (0.56 - 0.96)** | **0.45 (0.34 - 0.60)** | 0.81 (0.65 - 1.03) | 1.01 (0.79 - 1.28) | 0.77 (0.50 - 1.18) |
| **Activity patterns** |  |  |  |  |  |
| *Inactive* | 1 (ref) | 1 (ref) | 1 (ref) | 1 (ref) | 1 (ref) |
| *Weekend warrior* | **0.63 (0.48 - 0.84)** | **0.45 (0.33 - 0.61)** | **0.65 (0.51 - 0.82)** | 0.88 (0.68 - 1.13) | 0.83 (0.53 - 1.31) |
| *Regularly active* | **0.64 (0.48 - 0.86)** | **0.51 (0.38 - 0.68)** | **0.78 (0.62 - 0.99)** | 1.03 (0.81 - 1.32) | **0.55 (0.34 - 0.90)** |

Results are expressed as odds ratio (OR) and (95% confidence interval). Statistical analyses performed by logistic regressions adjusted for age, gender, professional occupation, educational level and accelerometer diurnal wear-time; with a further adjustment on body mass index ^1^. Significant (p<0.05) odds ratio are indicated in bold.

**Supplementary table 5:** Multivariate analysis of the cardiovascular risk factors associated with activity behaviours and patterns, excluding participants with history of cardiovascular disease. The CoLaus study, Switzerland, 2014-2017.

|  | **Smoking** | **Obesity** | **Hypertension ^1^** | **Dyslipidemia ^1^** | **Diabetes ^1^** |
| --- | --- | --- | --- | --- | --- |
| **Activity behaviours** |  |  |  |  |  |
| *Couch potato* | 1 (ref) | 1 (ref) | 1 (ref) | 1 (ref) | 1 (ref) |
| *Light mover* | 1.00 (0.68 - 1.46) | 1.01 (0.71 - 1.44) | 1.27 (0.90 - 1.78) | **1.49 (1.05 - 2.10)** | 0.97 (0.60 - 1.57) |
| *Sedentary exerciser* | **0.44 (0.29 - 0.67)** | **0.41 (0.27 - 0.62)** | 0.83 (0.60 - 1.15) | 1.23 (0.86 - 1.75) | 0.55 (0.30 - 1.01) |
| *Busy bee* | **0.66 (0.49 - 0.88)** | **0.42 (0.32 - 0.56)** | **0.78 (0.61 - 1.00)** | 1.09 (0.84 - 1.42) | **0.57 (0.38 - 0.86)** |
| **Activity patterns** |  |  |  |  |  |
| *Inactive* | 1 (ref) | 1 (ref) | 1 (ref) | 1 (ref) | 1 (ref) |
| *Weekend warrior* | **0.63 (0.46 - 0.86)** | **0.40 (0.29 - 0.56)** | **0.68 (0.52 - 0.89)** | 0.91 (0.68 - 1.21) | **0.50 (0.29 - 0.87)** |
| *Regularly active* | **0.60 (0.47 - 0.78)** | **0.42 (0.33 - 0.55)** | **0.74 (0.59 - 0.92)** | 0.99 (0.79 - 1.25) | **0.60 (0.41 - 0.87)** |

Results are expressed as odds ratio (OR) and (95% confidence interval). Statistical analyses performed by logistic regressions adjusted for age, gender, professional occupation, educational level and accelerometer diurnal wear-time; with a further adjustment on body mass index ^1^. Significant (p<0.05) odds ratio are indicated in bold.

**Supplementary table 6:** Multivariate analysis of the cardiovascular risk factors associated with activity behaviours and patterns, including all participants irrespective of missing data in cardiovascular risk factors. The CoLaus study, Switzerland, 2014-2017.

|  | **Smoking** | **Obesity** | **Hypertension ^1^** | **Dyslipidemia ^1^** | **Diabetes ^1^** |
| --- | --- | --- | --- | --- | --- |
| **Activity behaviours** |  |  |  |  |  |
| *Couch potato* | 1 (ref) | 1 (ref) | 1 (ref) | 1 (ref) | 1 (ref) |
| *Light mover* | 1.01 (0.72 - 1.44) | 0.98 (0.72 - 1.34) | 1.30 (0.95 - 1.77) | **1.52 (1.12 - 2.08)** | 0.94 (0.62 - 1.42) |
| *Sedentary exerciser* | **0.38 (0.25 - 0.57)** | **0.44 (0.31 - 0.64)** | 0.79 (0.58 - 1.08) | 1.11 (0.80 - 1.53) | **0.46 (0.26 - 0.81)** |
| *Busy bee* | **0.61 (0.47 - 0.80)** | **0.40 (0.31 - 0.52)** | **0.73 (0.58 - 0.92)** | 1.08 (0.85 - 1.37) | **0.61 (0.43 - 0.86)** |
| **Activity patterns** |  |  |  |  |  |
| *Inactive* | 1 (ref) | 1 (ref) | 1 (ref) | 1 (ref) | 1 (ref) |
| *Weekend warrior* | **0.57 (0.43 - 0.76)** | **0.40 (0.30 - 0.53)** | **0.66 (0.51 - 0.84)** | 0.89 (0.69 - 1.16) | **0.58 (0.37 - 0.92)** |
| *Regularly active* | **0.56 (0.44 - 0.71)** | **0.42 (0.33 - 0.53)** | **0.68 (0.56 - 0.83)** | 0.94 (0.76 - 1.15) | **0.59 (0.43 - 0.82)** |

Results are expressed as odds ratio (OR) and (95% confidence interval). Statistical analyses performed by logistic regressions adjusted for age, gender, professional occupation, educational level and accelerometer diurnal wear-time; with a further adjustment on body mass index ^1^. Significant (p<0.05) odds ratio are indicated in bold.

**Supplementary table 7:** Multivariate analysis of the cardiovascular risk factors associated with activity behaviours and patterns, without adjustment on body mass index. The CoLaus study, Switzerland, 2014-2017.

|  | **Hypertension** | **Dyslipidemia** | **Diabetes** |
| --- | --- | --- | --- |
| **Activity behaviours** |  |  |  |
| *Couch potato* | 1 (ref) | 1 (ref) | 1 (ref) |
| *Light mover* | 1.29 (0.95 - 1.76) | **1.43 (1.05 - 1.96)** | 0.96 (0.63 - 1.45) |
| *Sedentary exerciser* | **0.65 (0.48 - 0.88)** | 0.96 (0.70 - 1.33) | **0.40 (0.23 - 0.70)** |
| *Busy bee* | **0.61 (0.49 - 0.77)** | 0.90 (0.71 - 1.14) | **0.41 (0.29 - 0.58)** |
| **Activity patterns** |  |  |  |
| *Inactive* | 1 (ref) | 1 (ref) | 1 (ref) |
| *Weekend warrior* | **0.52 (0.41 - 0.66)** | **0.77 (0.59 - 0.99)** | **0.42 (0.26 - 0.66)** |
| *Regularly active* | **0.58 (0.48 - 0.71)** | **0.81 (0.66 - 1.00)** | **0.41 (0.30 - 0.58)** |

Results are expressed as odds ratio (OR) and (95% confidence interval). Statistical analyses performed by logistic regressions adjusted for age, gender, professional occupation, educational level and accelerometer diurnal wear-time. Significant (p<0.05) odds ratio are indicated in bold.
